# An Automobile’s Tail Lights: Sacrificing Safety for Playful Design?

**DOI:** 10.1177/00187208241237860

**Published:** 2024-03-15

**Authors:** Fiona Burns-Hemingway, Brett B. T. Feltmate, Raymond M. Klein

**Affiliations:** 1 3688 Dalhousie University, Halifax, Canada

**Keywords:** stimulus-response compatibility, driving safety, spatial Stroop, conflict resolution

## Abstract

**Objective:**

The counterintuitive “Union Jack”-inspired turn signals on versions of BMW’s Mini vehicles was investigated to reveal potential impacts on human performance.

**Background:**

When some Mini drivers indicate a change in direction, they do so with an oppositely oriented arrow. This conflict, between the task-irrelevant spatial shape and task-relevant location of the signal, mimics a “converse” spatial-Stroop effect that, in combination with the ubiquitous use of arrows on road signs, may be confusing.

**Method:**

Participants (*n* = 30) responded—via right and left keypresses—to the directions of road signs and turn signals in both pure and mixed blocks. Reaction times and accuracies were recorded to determine performance in each condition (compatible, neutral, incompatible).

**Results:**

Performance suffered when the location and direction of the stimuli did not correspond. When responding to turn signals the cost to performance was especially salient in mixed blocks. Thus, when driving on roads where the meanings of arrows on road signs is important, turn signals pointing in a direction opposite from the directional intention indicated by the signals’ location are likely to be confusing.

**Conclusion:**

The design of some Mini’s “Union Jack” style taillights opposes well-established principles of cognitive functioning, caused confusion in our laboratory study and therefore may be a safety hazard—a possibility that ought to be explored in more realistic (e.g., driving simulator) situations.

**Application:**

BMW designers should consider universally adopting the neutral, “horizontal line,” illumination style that is currently available in the aftermarket.

## Introduction

Designed by Sir Alec Issigonis of Morris Motors for the British Motor Corporation (BMC), the Mini became a British cultural icon shortly after its introduction circa 1960. Its mechanical (transverse engine, front wheel drive) innovations and spacious interior were design features that, along with fuel efficiency and excellent handling, would eventually lead to the Mini being named in 1999 “the second most influential automobile of the 20th century” and runner-up to Ford’s Model T (Wikipedia: https://en.wikipedia.org/wiki/Car_of_the_Century).

**Figure 1.**
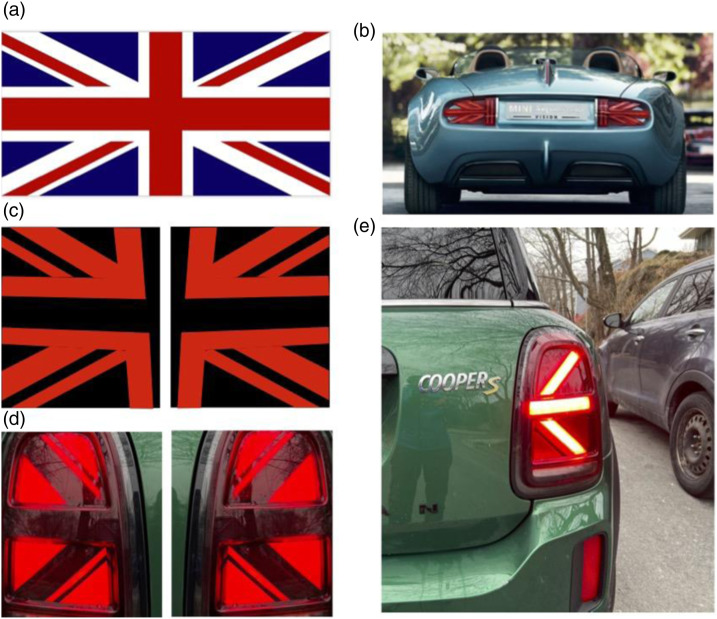
(a) British flag (Union Jack; from Flag of the United Kingdom by Smith, 2023, Encyclopædia Britannica Inc (https://www.britannica.com/topic/flag-of-the-United-Kingdom). Copyright 2022; used with permission.) (b) Based on the Union Jack, the 2014 design of the Mini Super Leggera concept car’s rear turn signals (Mini SuperLeggera Vision, n.d). This car was never manufactured, but the Union Jack inspired design became more explicit in later models that were widely sold beginning in 2018. (c) While the rear of the car separates the two signals, how this design reflects the Union Jack is easy to see by placing them close together. This illustration is based on an image of the taillight that can be found here: https://www.roehm.com/en/detail/mini-60-years-edition-union-jack-taillights-shine-brightly-thanks-to-plexiglas-molding-compounds. (d) A simplified design was later implemented. (e) An illustration of the conflict that is the topic of this paper: this driver is signalling that their car will be moving rightward but the arrow is pointing leftward. The car illustrated in D and E belongs to RMK.

This article is about an exterior design feature, the Mini’s tail lights, that was first imagined in 2014 by BMW Group (the German firm which had acquired the remains of BMC in 1996) to honour and reflect the car’s British heritage. The original design, based on the British “Union Jack” flag and its evolution are illustrated in Figure 1. Described as an “awesome,” “unique,” and “playful” design feature, some Mini owners without this feature could even acquire it with a retrofit accessory. Importantly, though, on many Mini models when a driver signals a rightward turn or lane change the right rear turn signal flashes a leftward pointing arrow (and vice versa). The possibility that this conflict might be unsafe, which can be found all over the internet (e.g.: https://teslamotorsclub.com/tmc/threads/mini-tail-lights.256907/, https://www.reddit.com/r/CrappyDesign/comments/oyywav/the_mini_cooper_countrymans_right_turn_signal_is/, https://jalopnik.com/congratulations-mini-you-made-the-stupidest-turn-sign-1847727385), was apparently dismissed by the car’s manufacturer: “…there should be no trouble at all for a driver to understand, when seeing the full rear of the car, which direction is being indicated” (as described here: https://jalopnik.com/congratulations-mini-you-made-the-stupidest-turn-sign-1847727385).

Although the conflict is undeniable, the dismissive view that it is the location and not the shape of the signal that informs us of the directional intentions of drivers, has merit. The purpose of the research project described here was to determine whether human performance is negatively affected by this conflict.

### Background

#### Road Safety

In 2018, the World Health Organization described road-traffic crashes as the eighth leading cause of death globally (WHO, 2018). Of the many factors involved in accidental motor-vehicle injuries, human error is considered to be overwhelmingly responsible (National Highway Traffic Safety Administration, 2008; Yau, 2004). In particular, a consistent link has been observed between road crashes and risky driving behaviours such as “turn signal neglect” (Nguyen-Phuoc et al., 2020). Turn signals are augmenting cues that provide road users with early and supposedly accurate information regarding drivers’ intentions to change directions (Mortimer, 1970). As such, to improve driver performance and increase road safety by reducing accidents, turn signal systems are both a required vehicle design feature (Moore et al., 1958) and their use by a driver to signal an intention to turn or change lanes is legally required. Given their behavioural relevance and the time pressures under which they are often interpreted, it is critical that turn signals be designed in a cognitively ergonomic fashion. Is the Union Jack tail light design of various Minis since 2018 such a poor design that it might be a safety hazard? We sought to answer this question by posing one that can be answered in the laboratory: “What occurs in an observer's mind when they see a vehicle signaling a directional change by an arrow pointing in the opposite direction?”

#### Cognitive Concepts

This is an applied question for which cognitive concepts such as stimulus-response (S-R) compatibility (e.g., Fitts & Seeger, 1953; Proctor & Reeve, 1989) and attentional set (e.g., Gibson, 1941; Leber & Egeth, 2006) are particularly pertinent. According to the S-R compatibility literature, incompatibility between the turn signal’s task-irrelevant spatial shape (i.e., arrow) and its task-relevant spatial location might impair one’s ability to interpret drivers’ intentions efficiently. Most readers will be familiar with the well-known Stroop effect (Stroop, 1935) which provides a powerful example of performance decrements in the face of such conflict. When the colour of ink is task-relevant the irrelevant word in which it is written interferes with colour naming, especially when the word is the name of a different colour. Importantly, the Stroop paradigm results in an asymmetric pattern of interference: the prepotent tendency to read interferes with colour naming while colours do not interfere much with reading.

The spatial nature of a car’s tail light turn signal and the Mini’s possibly untoward use of arrows place our project into the world of the spatial Stroop effect (e.g., Lu & Proctor, 1995). Spatial Stroop is an example of a Simon effect (e.g., Craft & Simon, 1970; later given this name by Hedge & Marsh, 1975) wherein the task-irrelevant location of a target interferes with spatial responses based on the target’s task-relevant identity. When the identity itself is spatial (e.g., an arrow or directional word) this has been called “spatial Stroop.” Like the Stroop effect, spatial Stroop is asymmetric: irrelevant location interferes with spatial responses based on arrow direction but arrow direction doesn’t interfere much with localization responses. Because of this asymmetric pattern of interference, one might imagine (as apparently BMW has asserted) that when interpreting the meaning of a vehicle’s turn signal, its task-irrelevant shape (e.g., conflicting arrow) might have no effect on performance.

Vehicle turn signals, however, are not perceived in isolation; they are generated and interpreted in the context of driving, wherein arrows on road signs often need to be attended, interpreted and sometimes responded to appropriately. According to the notion of attentional control settings, the ubiquitous use of arrows as directional markers on road signs should lead drivers to adopt an attentional set to interpret the shape/direction of arrows. Such a set—as activated in our mixed blocks—is expected to generate a converse spatial-Stroop effect (cf, Walley et al., 1994) wherein tail-light turn signals with incompatible arrows will generate costs in reaction time (RT) and/or accuracy relative to neutral signals.

## Experiment

Mental chronometry (Posner, 1978) was used to provide a window into the thought processes that might be activated by the aforementioned conflicts: With a focus on RT and accuracy, we asked participants in the laboratory to make keypress responses (left, right) to report the directions indicated by arrows on road signs and the directions indicated by tail light turn signals, all within the context of a mock highway display. For both target types, we manipulated the S-R compatibility (compatible, neutral, incompatible) between a target’s task-relevant (spatial shape for road signs, spatial location for tail light turn signals) and irrelevant (location for road signs, shape for turn signals) properties. Importantly, participants completed both “pure” (only one target type, turn signals or road signs) and “mixed” (equiprobable presentation of road sign or turn signal) blocks of trials.

### Predictions and Questions

Prediction 1: The ubiquitous spatial-Stroop effect will be observed when participants are responding to road signs in pure blocks (road signs only). More specifically, the compatibility between the task-irrelevant locations of road signs and the signs’ task-relevant directions, will affect both the speed and accuracy of responses.

Prediction 2: The aforementioned spatial-Stroop effect (Prediction 1) will, due to the increased importance of (set for) locations (of tail-light turn signals), be increased in the mixed blocks.

Question: Will a “converse spatial-Stroop” effect be observed when participants are responding to turn signals in pure blocks (turn signals only)? More specifically, will the compatibility between the task-irrelevant shapes of turn signals and the signals’ task-relevant location affect the speed and/or accuracy of responses?

Prediction 3: If observed in pure blocks with turn signals, the converse spatial-Stroop effect (Question) will be increased in mixed blocks due to an increased emphasis on (mental set for) road sign arrow directions. Even if such an effect is not observed in the pure blocks, it will be observed in the mixed blocks.

### Methods

This study was approved by Dalhousie University’s Social Sciences and Humanities Research Ethics Board and all participants provided proper informed consent. This study has been preregistered on the Open Science Framework (OSF; viewable at https://osf.io/vuqzh/).

#### Participants and Apparatus

Thirty participants recruited from the Dalhousie student body and general population (male = 10; median age = 21 and range = 19–38; all right-handed) took part in this experiment. Those tested in-person used their personal laptops in a designated testing room in the human research wing of the Psychology department, while remote participants were asked to use their laptops in a quiet room at home. The program controlling stimulus presentation and collection of responses was written in JavaScript using the experiment development framework, jsPsych (de Leeuw et al., 2023). Throughout the experiment, participants’ reaction times (ms) and accuracies (%) of responding were recorded and sent to a secure server at the Klein lab. The testing process was designed to take approximately 30 minutes in total and anonymized participant data is stored on the Open Science Framework repository.

#### Procedure

**Figure 2.**
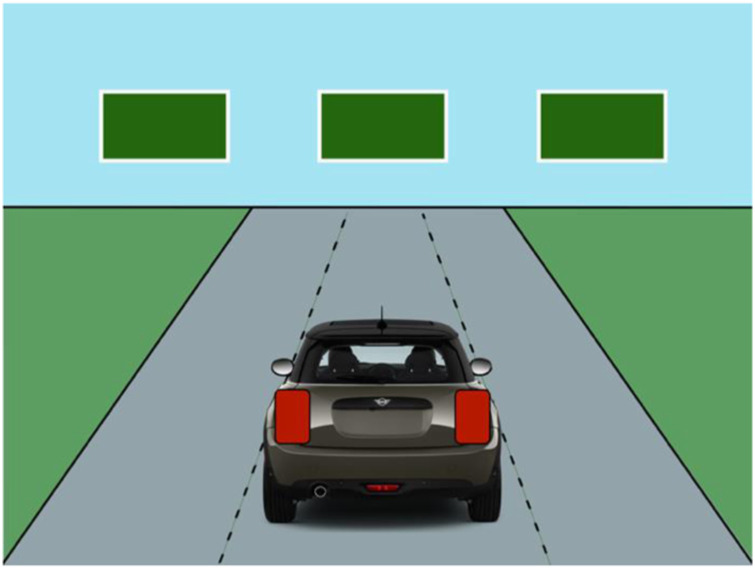
Base display prior to any target stimuli.

Trials began with a base display, with three spots for road sign stimuli at the top of the screen and the rear of a car with two places for turn signals at the bottom (see Figure 2).

After 500 ms, the target stimulus appeared (road sign or rear signal light), and the participant was tasked with indicating via a left or right key-press response, the direction of the arrow on a road sign or the location of the turn signal (depending on which target type was presented). Participants were instructed to do so as quickly as possible without sacrificing the accuracy of responding. If no response was registered within 1.5 seconds following target onset the trial self-terminated.

#### Design

Participants completed six blocks of trials: two consisting solely of turn signals (T), two consisting solely of road signs (R), and two consisting of both types of targets randomly intermixed (M). Testing sessions ended with the two mixed blocks, preceded by the four pure blocks, interleaved and counterbalanced across participants: RTRTMM or TRTRMM. Note that only one target type was ever presented on a particular trial, and regardless of block type the trial types composing it (see Table 1) were presented in unique random orders.

**Table 1.** Composition of the Three Types of Trial Blocks (Values Refer to the Total Number of Trials in a Block and the Numbers of a Specific Type of Trial Composing a Block).

			Type of Block		
Stimulus	Location	Location	Turn Signal (180)	Road Sign (180)	Mixed (360)
Left arrow	Left	Compatible	30	—	30
Left arrow	Right	Incompatible	30	—	30
Right arrow	Left	Incompatible	30	—	30
Right arrow	Right	Compatible	30	—	30
Plus sign	Left	Neutral	30	—	30
Plus sign	Right	Neutral	30	—	30
Left arrow	Left	Compatible	—	30	30
Left arrow	Right	Incompatible	—	30	30
Left arrow	Center	Neutral	—	30	30
Right arrow	Left	Incompatible	—	30	30
Right arrow	Right	Compatible	—	30	30
Right arrow	Center	Neutral	—	30	30

**Figure 3.**
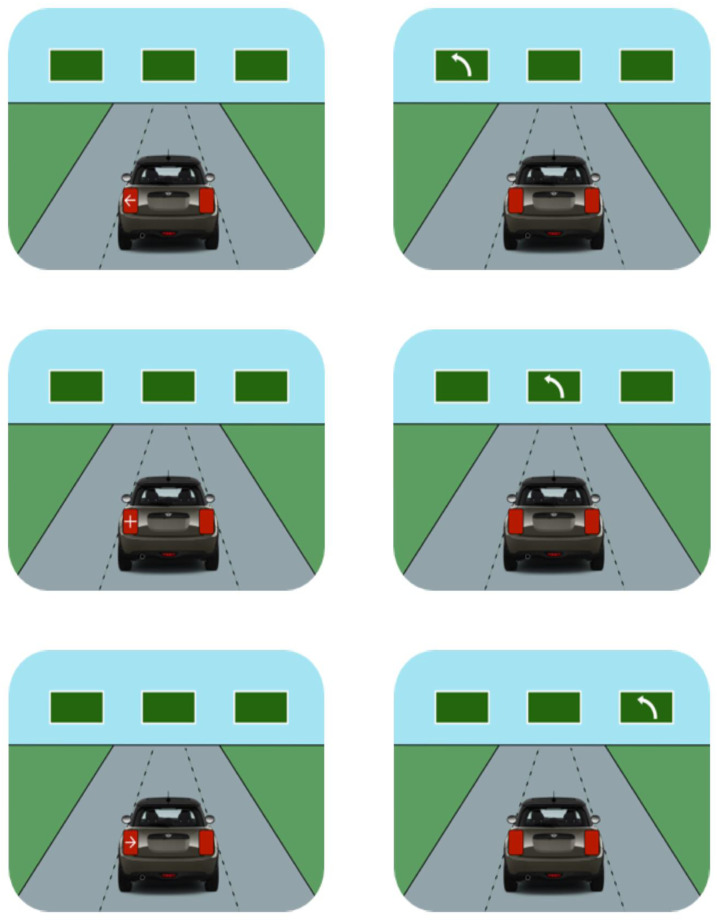
Six target types are illustrated. Turn signal targets are illustrated in the left column; road sign targets in the right column. Target condition is compatible in the top row; incompatible in the bottom row; neutral in the middle row. All targets represented here would require a left button press. The other 6 possible target types (all requiring right button press responses) consisted of the same turn signals illustrated on the left but in the right location and the opposite road signs (arrow) in the same locations.

Examples of some of the targets and their compatibility conditions are presented in Figure 3. Importantly, the bottom left target (rightward pointing arrow signalling a left turn) exemplifies the kind of incompatibility that—as described in the introduction—was incorporated into the design of some Mini’s rear turn signals (see Figure 1(e)).

#### Methods of Analysis

Prior to analysis, trials wherein participants failed to respond were removed (0.3% of trials). The remaining trial-by-trial data were trimmed using a cumulative accuracy function to exclude responses reflecting anticipatory or distracted behaviour, resulting in 1.3 % of trials being cut. The trimmed data were then examined to ensure that no participants disproportionately contributed to the excluded trials. Ultimately, the results from all 30 participants were included in the statistical analysis done using R software (version 4.2.1).

Using the Analysis of Factorial Experiments (afex) package, four 3 × 2-way repeated measures ANOVAs were conducted with stimulus compatibility (3 levels: compatible, neutral, incompatible) and block type (2 levels: pure, mixed) as predictors. This comprised of 3 × 2-way ANOVAs for both reaction time and accuracy of responding to road sign stimuli and to turn signal lights. Greenhouse-Geisser corrected degrees of freedom are provided for tests wherein the assumption of sphericity was found to be violated.

To estimate the effects of stimulus compatibility, planned comparisons in the form of t-tests (a priori *a* = 0.05) were conducted at two levels using the Estimated Marginal Means (emmeans) package. First, the main effects of incompatibility and compatibility were estimated by contrasting performance in those conditions against neutral (e.g., RT | incompatible − RT | neutral = incompatibility effect), which were performed separately for pure and mixed blocks. Second, because stimulus compatibility effects were expected to differ between block types, the above contrasts were then further contrasted to estimate the simple effect of block type (e.g., effect | pure − effect | mixed) on each. This procedure was conducted for both RT and error rates. RT analyses were only conducted on correct responses (∼93% of trials for road signs, ∼97% for turn signals).

### Results

**Figure 4.**
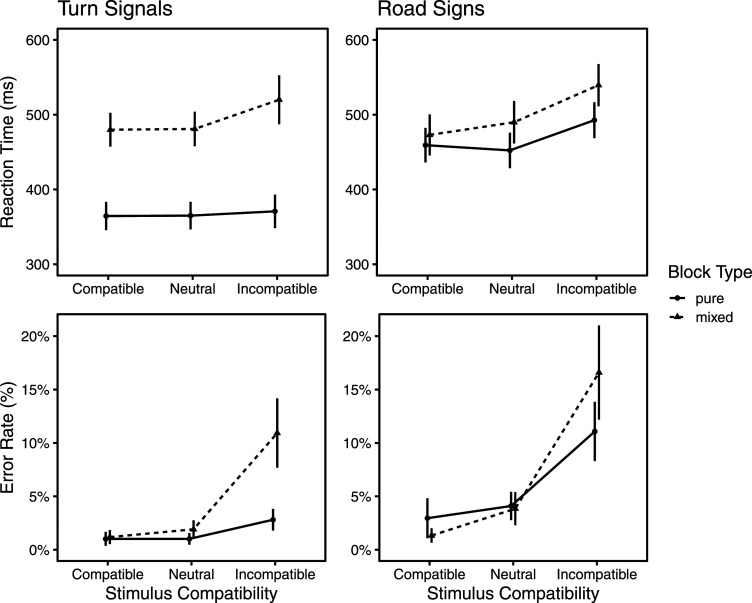
Reaction time and error rate by block type for turn signals and road signs. Error bars denote 95% CIs.

Performance (reaction time and accuracy) as a function of S-R compatibility, type of target (turn signals and road signs) and type of block (pure and mixed) is presented in Figure 4.

#### Turn Signals: Overall ANOVAs

In the analysis of RT, there were significant main effects of stimulus compatibility [*F* (1.22, 35.36) = 26.53, *p* < .001, $\eta_{G}^{2}$ = 0.029], and block type [*F* (1, 29) = 337.46, *p* < .001, $\eta_{G}^{2}$ = 0.511], which were qualified by a significant interaction effect [*F* (1.42, 41.23) = 25.69, *p* < .001, $\eta_{G}^{2}$ = 0.016]. Parallel effects were observed in the analysis of error rates, wherein the significant effects of stimulus compatibility [*F* (1.14, 33.03) = 53.05, *p* < .001, $\eta_{G}^{2}$ = 0.310] and block type [*F* (1, 29) = 24.80, *p* < .001, $\eta_{G}^{2}$ = 0.131] were qualified by a significant interaction [*F* (1.19, 34.40) = 29.57, *p* < .001, $\eta_{G}^{2}$ = 0.172].

#### Road Signs: Overall ANOVAs

In the analysis of RT, the significant main effects of stimulus compatibility [*F* (1.47, 42.51) = 95.12, *p* < .001, $\eta_{G}^{2}$ = 0.098] and block type [*F* (1, 29) = 23.12, *p* < .001, $\eta_{G}^{2}$ = 0.054] were qualified by a significant interaction effect [*F* (1.92, 55.60) = 20.05, *p* < .001, $\eta_{G}^{2}$ = 0.010]. In the analysis of error rates, stimulus compatibility was the only main effect [*F* (1.23, 35.72) = 58.57, *p* < .001, $\eta_{G}^{2}$ = 0.393]; however, there was a significant interaction [*F* (1.48, 43.04), *p* < .001, $\eta_{G}^{2}$ = 0.056] between stimulus compatibility and block type.

**Figure 5.**
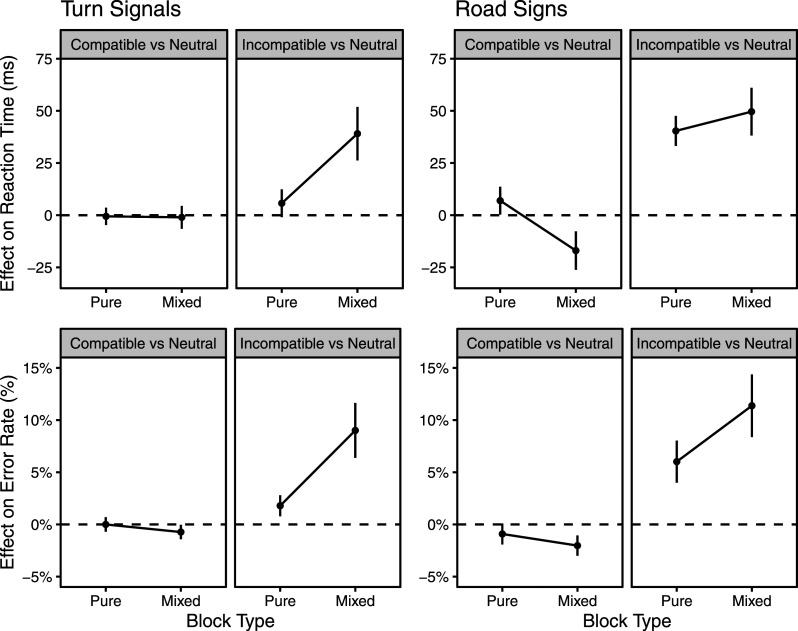
Contrasts assessing compatibility effects (relative to neutral). Positive values indicate performance costs. Error bars represent 95% CIs.

As noted above, planned contrasts (see Figure 5) were used to assess our predictions and questions.

#### Turn Signals: Planned Contrasts

In the pure blocks, neither compatible nor incompatible trials were significantly different in RT from neutral trials. Error rates, however, were significantly higher [*t* (29) = 3.63, *p* = .001, CI (0.008, 0.028)] in the incompatible condition (M = 2.8, *SD* = 2.7) than in the neutral condition (M = 1.0, *SD* = 1.4). In the mixed blocks, the incompatible condition (M = 520, *SD* = 88) was significantly slower [*t* (29) = 6.23, *p* < .001, CI (26.26, 51.91)] than the neutral condition (M = 480, *SD* = 62). Reinforcing this difference, responses in the incompatible condition (M = 10.9, *SD* = 8.6) were significantly [*t* (29) = 7.01, *p* < .001, CI (6.4, 11.6)] less accurate than those in the neutral condition (M = 1.9, *SD* = 2.2). Incompatibility costs to RT were significantly greater [*t* (29) = −5.73, *p* < .001, CI (−45.3, −21.4)] in mixed blocks (M = 39.1, *SD* = 34) than in pure blocks (M = 5.7, *SD* = 17.8). Likewise, incompatibility costs to accuracy were greater [*t* (29) = −5.40, *p* < .001, CI (−0.10, −0.04)] in mixed blocks (M = 9.0, *SD* = 7.0) compared to pure blocks (M = 1.8, *SD* = 2.7).

#### Road Signs: Planned Contrasts

In the pure blocks, there were no benefits on compatible (relative to neutral) trials but there were significant costs in both RT and accuracy when incompatible trials were compared to neutral trials: incompatible RT (M = 492, *SD* = 64) was significantly [*t* (29) = 11.49, *p* < .001, CI (33.21, 47.58)] slower than neutral RT (M = 452, *SD* = 64); and reinforcing this difference, incompatible error rate (M = 9.3, *SD* = 7.1) was significantly higher [t (29) = 5.85, *p* < .001, CI (8.4, 14.4)] than neutral error rate (M = 3.7, *SD* = 3.1). In the mixed blocks, significant benefits emerged for both RT and accuracy: in other words, compatible RT (M = 472, *SD* = 73) was significantly [t (29) = −3.76, *p* < .001, CI (−26.14, −7.75)] faster than neutral RT (M = 489, *SD* = 76) and compatible error rate (M = 1.0, *SD* = 1.4) was significantly lower [t (29) = −4.07, *p* < .001, CI(−3.00, −1.05)] than neutral error rate (M = 3.1, *SD* = 3.0). The significant costs seen in pure blocks were preserved in the mixed blocks: Incompatible RT (M = 540, *SD* = 75) was significantly [t (29) = 8.87, *p* < .001, CI(38.18, 61.06)] slower than neutral RT (M = 489, *SD* = 76); and reinforcing this difference, incompatible error rate (M = 14.4, *SD* = 10.0) was significantly higher [t (29) = 7.43, *p* < .001, CI(8.4, 14.4)] than neutral error rate (M = 3.1, *SD* = 3.0). Mathematically speaking, incompatibility costs to RT were greater in mixed (M = 49.6, *SD* = 30.6) than in pure (M = 40.4, *SD* = 19.2) blocks, but this difference did not survive testing [*t* (29) = −1.56, *p* = .128, CI (−21.3, 2.8)]. For error rates, however, incompatibility costs were significantly [*t* (29) = −4.16, *p* < .001, CI (−7.9, −2.8)] higher in mixed (M = 11.4, *SD* = 8.4) than in pure (M = 6.0, *SD* = 5.6) blocks.

### Discussion

These results confirm the three predictions outlined in our introduction and provide a clear answer to the questions posed there. For responses to on-road directional-signs, we replicated the “classic” spatial-Stroop effect, wherein participants were on average slower and more error-prone when the location of the road sign did not correspond with the indicated direction (H1). This effect was observed in both pure and mixed blocks, with evidence suggesting that costs were exacerbated in mixed blocks (H2). For responses to turn signals, and answering our question, we observed a small, but significant, “converse” spatial-Stroop effect in accuracy in the pure blocks. Importantly, in the mixed blocks participants were both slower and more error-prone when responding to turn signals when the task-irrelevant shape of these signals did not correspond with their task-relevant location (H3). This demonstrates that the Union Jack-based tail light design of various Minis since 2018 was a poor design choice and a possible safety hazard.

Whereas keypresses are not directly analogous to “real” driving behaviours, how a driver reacts to turn signals is a consequence of their internal perception of such signals, which we believe we have measured here. Taken together, our results provide decisive evidence that the “Union Jack” style tail-light design is at odds with principles of cognitive functioning which have been well-known and well-demonstrated for over half a century.

## Limitations and Future Directions

It appears that road signs conveying relevant directional information via arrows are necessary to robustly reveal possible safety implications of the Mini’s Union Jack-inspired design. However, other practically relevant distractions—including pedestrians, passing vehicles and road hazards—were not accounted for in our task. Thus, the results from our mock road sign and turn signal laboratory study are preliminary and may not generalize to the context of actual driving performance. That noted, one might infer that the additional cognitive demands imposed by real driving might increase the degree to which arrow turn signals are automatically processed (Galfano et al., 2012; Tipples, 2002) thereby exacerbating the costs above what we have observed in our nondriving task. Further research with greater ecological validity (e.g., using a driving simulator, Meuleners & Fraser, 2015) is necessary to determine the actual degree to which the Mini’s counter-intuitive rear signal lights might be dangerous in the real world.

## Conclusion

**Figure 6.**
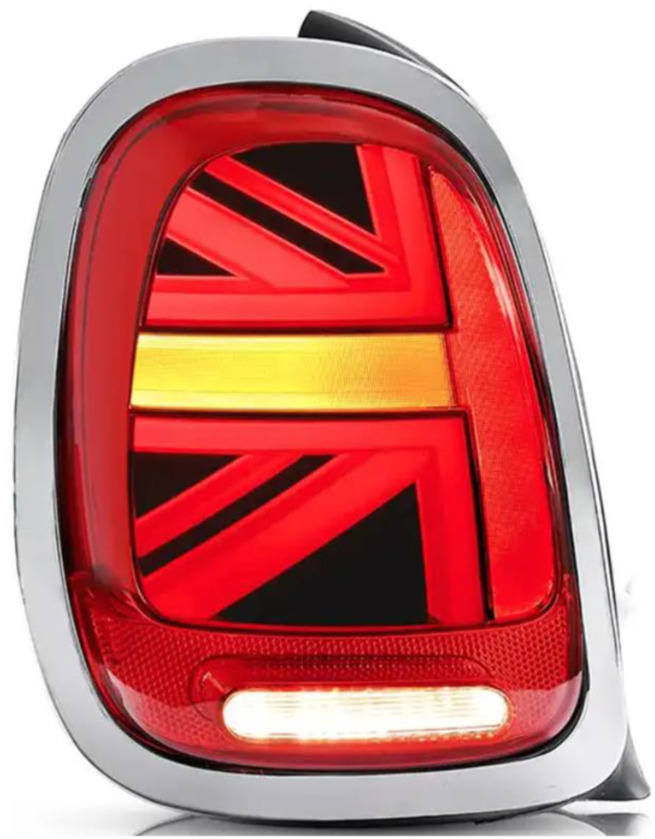
Union Jack-inspired taillight design that likely avoids the safety hazard implied by the research reported here. Instead of illuminating an arrow to signal a lane change or turn, here the horizontal element of the design is simply illuminated. (Vland LED Tail Lights, n.d).

We believe that BMW needn’t abandon its playful Union Jack design because there is a simple way to avoid the possible safety hazard demonstrated here. Instead of illuminating an arrow when the driver is signalling a turn or lane change, just illuminate the horizontal line that is a component of the Union Jack design. Precisely this design choice has been seen on some Mini models (see Figure 6). If BMW agrees with the implications of our research perhaps they will universally implement this “horizontal line” design choice.

As we see it, the playful design choice to reflect the Union Jack in the taillights of cars in the Mini family, when combined with the flashing arrow as its turn signal, represents a failure to consider basic principles of human performance (e.g., Fitts & Posner, 1967). The importance of these principles was engagingly and skillfully illustrated in two editions of Donald Norman’s books on the psychology and design of everyday things (Norman, 1988, 2013). One notable, and general, message of the research presented here is that engineers and designers of devices that humans interact with should pay attention to these principles to maximize safety, efficiency and comfort.

## Key Points

- Replicating the “classic” spatial-Stroop effect, responses reporting the direction signalled by road signs were slower and less accurate when the signs’ task-irrelevant locations conflicted with their indicated directions,
- Representing a “converse” spatial-Stroop effect, when reporting the location of turn-signals, responses were slower and less accurate when signals’ task-irrelevant shapes conflicted with their locations.
- These negative effects on performance were generally increased in mixed blocks wherein road signs and turn signal targets were randomly intermixed.
- Inward-facing arrows were used as turn-signals to visually mimic the “Union Jack” rear-light design on many models in the Mini line of vehicles.
- The pattern of costs observed here suggests that this design invokes cognitive conflict and is possibly a safety hazard.
